# Impacts of Liver Resection with Neoadjuvant Treatment Versus Surgery Alone in Hepatocellular Carcinoma with Portal Vein Tumour Thrombosis: A Systematic Review and Meta-Analysis

**DOI:** 10.3390/cancers18020277

**Published:** 2026-01-16

**Authors:** Poyyamozhi Rajagopal, Kesav Aditya Vijayagopal, Shanmuga S. Kannan, Shraddha Shetty, Madhava Pai

**Affiliations:** 1 Department of HPB Surgery, Hammersmith Hospital, Imperial College Health Care NHS Trust, London W12 0HS, UK; 2 Department of UGI Surgery, Luton & Dunstable University Hospital, Luton LU4 0DZ, UK; shanmuga.kannan2@nhs.net; 3 Department of Surgery & Cancer, Imperial College London, London SW7 2AZ, UK

**Keywords:** hepatocellular carcinoma (HCC), portal vein tumour thrombosis (PVTT), Barcelona Clinic Liver Cancer Staging C (BCLC Stage C), Trans Arterial Chemo Embolisation (TACE), Hepatic Artery Infusion of Chemotherapy (HAIC), Tyrosine Kinase inhibitors (TKI), PD-1 inhibitors, hepatectomy

## Abstract

**Background**: Hepatocellular carcinoma (HCC) with portal vein tumour thrombosis (PVTT) has a poor prognosis, and the benefits of neoadjuvant therapy are unclear. This systematic review and meta-analysis aim to evaluate the impact of neoadjuvant treatment (NAT) followed by surgery versus surgery alone on survival outcomes. **Methods:** A PRISMA-compliant systematic review was conducted by searching the OVID databases Embase, Medline, PubMed, and Scopus for English-language comparative studies of resectable HCC with PVTT, up to 23 January 2025. Two reviewers independently screened, extracted data, and assessed risk of bias (ROBINS-I/ROB2). Hazard ratios (HRs) for overall survival (OS) and recurrence-free survival (RFS) were pooled for meta-analysis. **Results:** Seven studies (2015–2024, five retrospective cohorts, one non-randomised comparative, one RCT) included 621 patients. The pooled analysis demonstrated that NAT followed by surgery was associated with a significantly improved OS (HR: 0.48, 95% CI: 0.295–0.67, *p*-value < 0.001, I^2^ = 0.00) and improved RFS (HR: 0.4, 95% CI: 0.2–0.58, *p*-value < 0.001, I^2^ = 0.00). **Conclusions**: For patients with HCC and an associated PVTT, neoadjuvant treatment before surgery significantly improves both overall and recurrence-free survival. These findings support a multimodal approach. Current evidence is largely non-randomised and HBV-endemic, warranting prospective validation in aetiologically diverse cohorts, including Western ones.

## Simple Summary

Hepatocellular carcinoma (HCC) with portal vein tumour thrombosis (PVTT) is a well-known complication of HCC and is associated with a dismal prognosis, with minimal treatment options. According to the Barcelona Clinic Liver Cancer (BCLC) staging system, HCC with PVTT represents an advanced stage, where systemic therapy is typically the primary treatment. And the role of liver resection after neoadjuvant treatment in this challenging patient group remains unclear. This systematic review and meta-analysis evaluated outcomes of liver resection following neoadjuvant therapy compared with surgery alone in patients with hepatocellular carcinoma (HCC) associated with portal vein tumour thrombosis (PVTT). Pooled data showed that neoadjuvant therapy before resection was associated with improved overall and recurrence-free survival compared with surgery alone. These findings support a combined modality approach in selected patients with HCC and PVTT and highlight the need for future multicentre randomised controlled trials to establish the optimal treatment strategy for this challenging patient group.

## 1. Introduction

Hepatocellular carcinoma (HCC) accounts for approximately 85% of primary liver cancers and contributes substantially to global cancer mortality; in 2022, liver cancer ranked as the third leading cause of cancer death worldwide, accounting for ~7.8% of all cancer deaths [1,2]. The global burden of HCC is projected to increase, with incidence expected to rise more than 55% between 2020 and 2040 [3]. Together with persistent challenges in early detection—nonspecific symptoms until advanced stages, slow tumour progression, and limitations in current surveillance—this is likely to result in a larger pool of patients presenting with advanced HCC [4,5]. Portal vein tumour thrombosis (PVTT) represents a common manifestation of advanced HCC and is identified in 10% to 40% of patients at initial diagnosis [6,7]. Beyond reflecting aggressive tumour biology and high tumour burden, PVTT exacerbates mortality by precipitating portal hypertension and its complications (e.g., oesophago-gastric varices, haemorrhage, and refractory ascites) and is associated with poor survival, with a median survival of 2 to 4 months in untreated patients [8,9]. While Barcelona Clinic Liver Cancer (BCLC) guidelines generally direct PVTT cases toward systemic therapy, increasing evidence and technical advances in hepatic surgery and locoregional/neoadjuvant treatments support curative intent approaches for appropriately selected patients with a 5-year survival rate of 39.1% to 55.9% [10,11]. Accordingly, when curative intent is pursued, the optimal sequence remains debated: upfront hepatectomy (with thrombectomy and/or vascular control/reconstruction where required) versus neoadjuvant/downstaging therapy followed by resection [12]. Upfront surgery may facilitate prompt R0 resection in technically resectable disease, whereas neoadjuvant approaches may reduce tumour/thrombus burden, address occult micrometastatic disease, and refine selection based on tumour biology but may also risk progression, toxicity, and deterioration in liver function that can preclude resection [13,14,15]. In practice, sequencing is individualised within a multidisciplinary framework, based on PVTT extent, resectability, future liver remnant, baseline liver function/portal hypertension, performance status, and response to initial therapy.

This systematic review and meta-analysis aim to synthesise the available evidence on neoadjuvant and downstaging strategies delivered prior to hepatectomy in patients with HCC and PVTT and to assess whether preoperative therapy improves resectability and pathological response and translates into clinically meaningful oncological benefit (overall and recurrence-free survival) without incurring unacceptable perioperative morbidity. By delineating the balance of potential benefits and risks, this review seeks to inform multidisciplinary decision-making regarding patient selection and to clarify when a neoadjuvant-first strategy may be favoured over upfront surgery.

## 2. Materials and Methods

**Figure 1:**
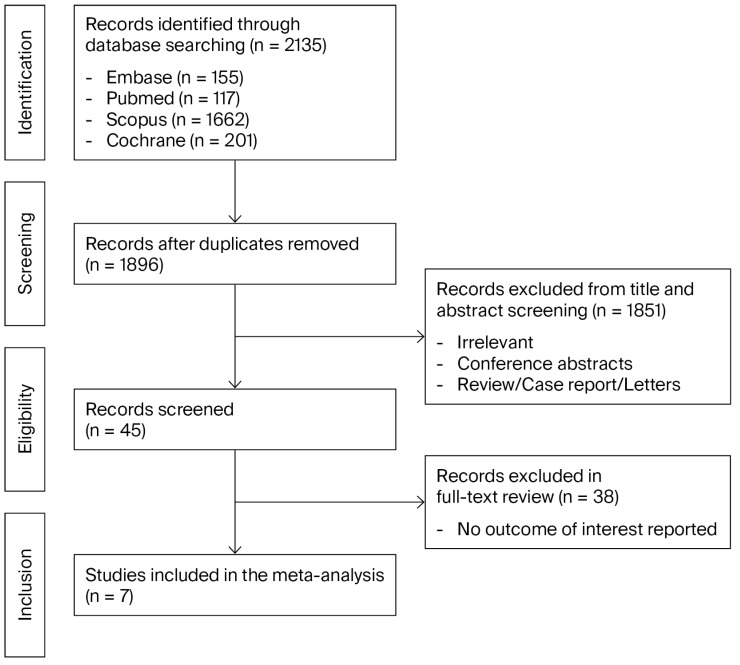
PRISMA flow diagram.

This review was performed in accordance with the PRISMA (Preferred Reporting Items for Systematic Reviews and Meta-Analyses) guidelines [Figure 1] and has not been registered. Neither an institutional review board approval nor informed consent was required for this review.

### 2.1. Search Strategy

A systematic search was independently conducted by two authors for published studies up to the 23 January 2025, using open-ended MeSH terms across the Medline, PubMed, Cochrane, Embase, and Scopus databases to identify all the published literature. The search string used was (“Hepatocellular carcinoma” OR “HCC” OR “hepatocellular carcinoma”) AND (“Portal Vein Tumour”). The corresponding references were screened manually. No other restrictions were applied to the systematic search beyond English-language publication.

### 2.2. Eligibility Criteria

Two authors independently reviewed and screened all retrieved studies by title and abstract. Once this was completed, the full texts of each of the filtered lists were reviewed for inclusion based on (1) patient population of interest: patients diagnosed with resectable HCC and PVTT at any stage based on Cheng’s Classification or Japanese Classifications; (2) interventions: Neoadjuvant treatments such as Hepatic Artery Infusion of Chemotherapy (HAIC), Tyrosine Kinase Inhibitors (TKIs), Trans Arterial Chemo Embolisation (TACE), Radiotherapy, 3D Conformational Radio therapy (3DCRT) together with a surgical resection, or PD-1 Inhibitors; (3) outcomes: at least one clinical outcome of interest, including overall survival (OS) or recurrence-free survival (RFS) with reported hazard ratios (HRs). Studies were excluded based on the following: (1) studies describing patients deemed unresectable; (2) patients undergoing adjuvant treatments only with no neoadjuvant treatments; (3) conference abstracts, reviews, editorial reviews, case reports, and expert opinions.

### 2.3. Data Extraction

All text, figures, and tables in the filtered articles were reviewed, and the parameters of interest were extracted using a pre-designed data extraction form, which included (1) first author; (2) year of publication; (3) neoadjuvant treatments performed; (4) control population; (5) number of patients in the control; (6) number of patients in the treatment groups; (7) Child–Pugh Grade of patient population; (8) Cheng Stage where reported; (9) Japanese stage of PVTT; (10) median follow-up time; (11) median RFS at 1, 3, and 5 years for intervention and control groups; (12) median OS at 1, 3, and 5 years; (13) confidence intervals; (14) hazard ratio (HR); and (15) odds ratio (OR) where HR were not reported. For articles that reported OR only, HR was indirectly computed. For articles that did not report HR or OR values, the HR was indirectly extracted from the corresponding survival estimates in the presented Kaplan–Meier curves using Engauge Digitiser software version 12.1 and calculated according to the methods described by Tierney et al. [16].

### 2.4. Risk of Bias Assessment

Two reviewers independently assessed the risk of bias and methodological quality. Discrepancies were resolved through discussion, with adjudication by a third reviewer where agreement could not be reached.

The risk of bias in non-randomised studies was assessed by the ROBINS-I tool, which evaluates seven domains such as confounding bias, selection bias, bias due to classification of interventions, bias due to deviations from intended interventions, bias due to missing data, measurement of outcomes bias, and bias due to selection of the reported results Table 1 [17]. Each study was classified as having low, moderate, serious, or critical risk of bias or as providing no information for a given domain. Methodological quality of observational studies was assessed using the Newcastle–Ottawa Scale (NOS), evaluating selection, comparability, and outcome/exposure domains, with studies categorised as being of good, fair, or poor quality [18].

The quality of the one randomised controlled trial included was evaluated using the Cochrane Risk of Bias (ROB2) tool to assess methodological quality Table 2 [25]. This examined randomisation techniques, deviations from the intended interventions, missing data, outcome measures, and the selection of report items for all included randomised controlled trials. Each of the randomised controlled trials was ranked according to its assessment outcome as “low-risk”, “high-risk”, or “some concerns”.

### 2.5. Statistical Analysis

For both overall survival (OS) and recurrence-free survival (RFS), a meta-analysis was conducted using a random-effects model, Hedges’ effect size estimate, and inverse-variance weighting. Significance was set at *p* < 0.05, and all *p*-values were two-tailed. All analyses were performed using IBM SPSS Statistics, Version 29.0.2.0.

## 3. Results

The initial database search resulted in 2135 relevant articles. Of these, 239 duplicate articles were excluded, yielding 1896 articles for screening of titles and abstracts. After screening for titles and abstracts, 45 articles were eligible for full-text review. After reviewing the full text of each identified article, seven were included in the final meta-analysis.

The main characteristics of the included studies are summarised in Table 3. All studies were published between 2015 and 2024. Among the seven articles, five were retrospective cohort studies, one was a randomised controlled trial, and one was a non-randomised comparative study. The number of patients ranged from 20 to 138 across the studies, yielding a total of 621 patients identified in this meta-analysis. A total of 275 patients received neoadjuvant treatment compared to 346 patients identified as undergoing surgery without neoadjuvant therapy. Neoadjuvant treatments included Tyrosine Kinase Inhibitors (TKIs), PD-1 inhibitors, Trans-Arterial Chemo-Embolisation (TACE), and Hepatic Artery Infusion of Chemotherapy (HAIC).

### Results of the Traditional Pairwise Meta-Analysis

**Figure 2:**
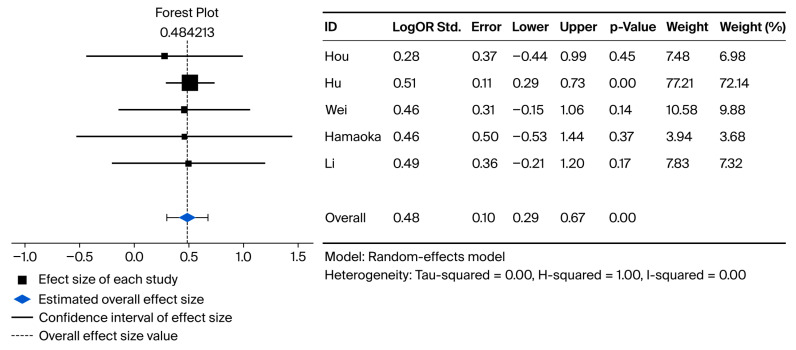
Forest plot representing pairwise meta-analysis between neoadjuvant treatment + liver resection versus liver resection alone for overall survival [19,21,22,23,26].

**Figure 3:**
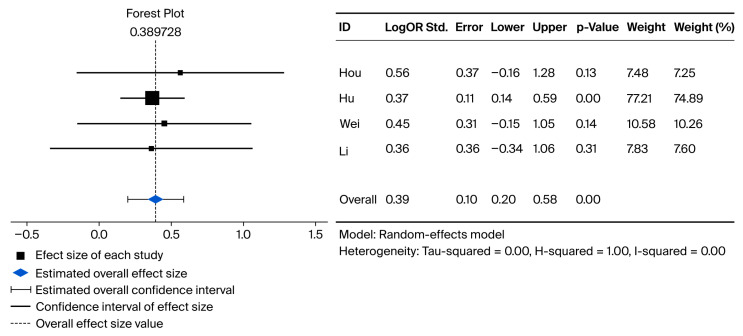
Forest plot representing pairwise meta-analysis between neoadjuvant treatment + liver resection and liver resection alone for recurrence-free survival [19,21,23,26].

As described previously in the statistical plan, a random-effects model was employed to pool the results. Full data were available for pooled meta-analysis for five studies in OS and for four studies in RFS. Compared with direct-to-surgery treatment strategies, neoadjuvant treatment + surgery showed significantly improved overall survival and recurrence-free survival, with logs of HRs of 0.48 (95% CI: 0.295–0.67, *p*-value: <0.001, I^2^ = 0.00) and 0.4 (95% CI: 0.2–0.58, *p*-values: <0.001, I^2^ = 0.00), respectively. The Forest plots for the pairwise meta-analysis of OS and RFS are shown below in Figure 2 and Figure 3, respectively.

**Figure 4:**
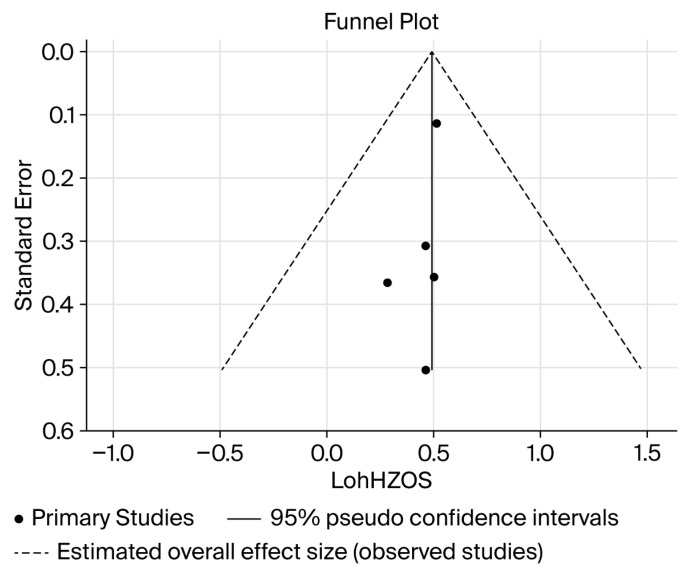
Funnel plot representing publication bias among included studies between neoadjuvant treatments + liver resection versus liver resection alone for overall survival. OSHZ = overall survival hazard ratio.

A funnel plot (Figure 4) shows the point of no effect for the intervention provided for overall survival and recurrence-free survival. The funnel plots are symmetrical, suggesting a low risk of publication bias across the included studies. All reported studies lie close to the central effect line, with no statistically significant outliers for either overall survival or recurrence-free survival.

## 4. Discussion

Hepatocellular carcinoma (HCC) with portal vein tumour thrombosis (PVTT) combines technical complexity with high-systemic-risk biology. For neoadjuvant strategies, a key consideration is not only which treatment can produce radiological response but which approach is deliverable and potentially beneficial for a given PVTT extent and hepatic reserve while preserving the opportunity for curative intent surgery. As the evidence base remains heterogeneous (with limited randomised data and substantial selection effects), any cross-study inferences should be framed as hypothesis-generating. In that context, because each modality has been applied to a different anatomical spectrum of PVTT, outcomes can be interpreted through a preoperative selection framework that considers (1) Cheng/vp extent, (2) differential response of primary tumour versus thrombus, (3) monitoring and non-response, (4) modality-specific toxicity constraints, and (5) the observed direction and magnitude of effects on OS and DFS/RFS.

Wei et al. randomised patients with Cheng II–III PVTT (*n* = 164; Cheng II 92, Cheng III 72) to neoadjuvant RT followed by surgery versus surgery alone, and the reported radiologic response was 20.7% for the primary tumour and downstaging of PVTT occurred in 12/82 (14.6%) of the RT arm [26]. Li et al. focused on the main PVTT (Cheng III) using neoadjuvant RT before hepatectomy. In Li et al., PVTT appeared more radiosensitive than the primary tumour (PVTT partial response (PR) 26.7% vs. tumour PR 13.3%), with downstaging from Cheng III to II in 6/45 patients [23]. Using the Japanese system, Yamamoto et al. show that although vp3–4 represents more proximal PVTT, carefully selected patients undergoing hepatectomy after TACE can achieve survival comparable to vp1–2 (5-year OS 30.0% vs. 47.2%; 5-year DFS 20.0% vs. 19.6%) [24]. However, they highlight that portal obstruction can limit the efficacy of embolisation-based therapy, supporting the broader premise that approaches that preserve or restore portal patency may expand downstream treatment options. Hu et al. compared PVTT extent using the Japanese system (Vp1/2 vs. Vp3/4), and outcomes were reported stratified by this extent [21]. In the neoadjuvant FOLFOX-HAIC cohort, the primary tumour response rate by mRECIST exceeded 50%, with CR 10.8% (7/65) and PR 49.2% (32/65). However, PVTT-specific radiological response was not reported in a parallel complete response (CR)/partial response (PR)/stable disease (SD)/progressive disease (PD) table. Consequently, thrombus control is inferred indirectly from VP-extent stratification and the operability requirement that all disease, including PVTT, remains technically resectable. These results suggest single-modality neoadjuvant approaches could potentially improve either tumour control or thrombus-related feasibility.

Multiple studies have shown that adding a second modality to a single-modality backbone consistently improves PVTT control, often with parallel gains in time-to-progression and/or overall survival compared with the single-modality approach [27,28,29,30,31]. Hou et al. evaluated the efficacy and safety of locoregional–systemic neoadjuvant therapy (TACE + TKI + PD-1) before resection in branch PVTT (Cheng type I–II) [19]. In this cohort, deep primary tumour responses were reported (complete response (CR) 39.4%, partial response (PR) 39.4%, stable disease (SD) 21.2%) alongside a meaningful pathological signal (pCR 27.2%) and substantially lower recurrence than surgery alone (11/33 [10.5%] vs. 80/105 [76.2%]). These findings support the concept that, when portal inflow is preserved, multimodality neoadjuvant therapy can improve both the intrahepatic tumour compartment and thrombus biology before surgery. However, interpretation should note that Hou et al. incorporated protocolised postoperative treatment (maintenance systemic therapy ± additional locoregional therapy), whereas in the other surgical cohorts, postoperative therapy was generally described as salvage/palliative treatment given at the time of recurrence rather than a uniform adjuvant strategy [19]. Taken together, these “single vs. double” comparisons support the rationale that PVTT is frequently the rate-limiting target and that the neoadjuvant strategy should be conceptualised as a staged, combined-treatment pathway, by first securing thrombus/portal flow control and escalating liver-directed and systemic components to address intrahepatic burden and micrometastatic risk. In advanced PVTT (Vp3/Vp4), Hamaoka et al. combined HAIC with PVTT-targeted 3D-CRT and reported that thrombus response was achievable in a substantial proportion (PVTT ≥ TE3: 32/52, 61.5%), and among seven patients who ultimately underwent resection, response of PVTT and main tumour were observed in seven and five patients, respectively [22]. Jiao et al. used systemic combination therapy with a TKI and PD-1 in Vp3/Vp4 patients prior to salvage surgery and observed a complete response (CR) in 15.0% (6/40) and a partial response (PR) in 77.5% (31/40) of patients [20]. Interestingly, there was the apparent discordance between radiological and pathological assessment of thrombus response as PVTT downstaging on imaging occurred in 37.5% (15/40) (Vp3 36.4%, Vp4 38.9%). In contrast, pathological evaluation demonstrated PVTT pathological complete response (pCR) in 62.5% (25/40) (Vp3 63.6%, Vp4 61.1%), suggesting that imaging criteria may underestimate thrombus sterilisation in selected surgical cohorts.

Because deliverability influences real-world effectiveness, treatment monitoring and explicit definitions of non-response are essential. Across studies, monitoring addresses two practical questions: whether a viable tumour is controlled (typically via contrast CT/MRI and mRECIST) and whether the thrombus is controlled sufficiently to permit safe resection (PVTT extent/flow with reassignment of Cheng/Japanese stage where feasible). Biomarkers such as AFP dynamics are commonly used as adjuncts, and Hu et al. [21]. additionally leveraged inflammatory biology (CRP) in predictive modelling. Physiological monitoring—bilirubin and synthetic function, ascites, portal hypertension/varices, FLR assessment, and operability review—is central to maintaining a surgical window. Predictive modelling can help operationalise selection and reduce futile treatment. The authors proposed a logistic regression model using baseline AFP and CRP to predict HAIC response (AUC 0.756), with a probability cut-off of 0.468 yielding sensitivity of 0.722 and specificity of 0.724. This supports a broader principle relevant across modalities: response-adapted pathways and biomarker-informed selection can improve deliverability by concentrating neoadjuvant exposure in patients most likely to achieve meaningful downstaging or tumour control.

A particular challenge at the monitoring stage is interpreting stable disease (SD). This finding in mRECIST may still represent a clinically useful outcome if it is accompanied by PVTT control (e.g., reduced extent, improved portal flow, or no further proximal progression) and preservation of liver reserve, thereby maintaining a safe window to proceed to the planned hepatectomy [20,26]. Conversely, stable disease without thrombus improvement, when coupled with rising AFP, worsening liver function, could suggest inoperable disease [21]. Against this background, non-response rates vary by modality and are best expressed using two complementary denominators: radiologic non-response (typically SD/PD) and clinical non-response (failure to proceed to the planned hepatectomy). In Wei et al., radiologic non-response was common (SD 58/82 [70.7%] and progressive disease (PD) 7/82 [8.5%], i.e., SD/PD 65/82 [79.3%]), while clinical non-response (“failure to reach surgery”) occurred in 9/82 (11.0%) after RT [26]. In Li et al., non-response differed by compartment: for the primary tumour, SD/PD was 39/45 (86.7%), whereas for PVTT, SD/PD was 33/45 (73.3%), and 6/45 (13.3%) developed contraindications to hepatectomy after RT [23]. In Hu et al., primary tumour non-response by mRECIST was lower (SD 24/65 [36.9%] and PD 2/65 [3.1%], i.e., SD/PD 26/65 [40.0%]), and an intention-to-treat view adds attrition prior to surgery (6/71 [8.5%] who started HAIC did not reach hepatectomy) [21].

Across the included studies, peri-treatment toxicity was generally manageable within the selected surgical candidates, but the pattern of complications differed by modality and may help explain attrition before hepatectomy. RT-based neoadjuvant pathways were characterised by infrequent but potentially pathway-limiting hepatic events like liver toxicity, HBV reactivation, and liver function deterioration [23,26]. Among those who proceeded to hepatectomy, postoperative morbidity was largely comparable to the surgery-only cohort. HAIC-based neoadjuvant therapy was associated predominantly with transient hepatic enzyme elevation and hematologic toxicity (leukopenia 34.3%, thrombocytopenia 32.9%), and postoperative complications and 90-day mortality were similar to upfront surgery (4.6% vs. 5.5%) [21]. Locoregional–systemic ‘triple therapy’ produced frequent but mostly low-grade treatment-related adverse events and postoperative morbidity after matching was comparable to surgery-only controls with 27.6% overall complications (Clavien–Dindo III–IV 8.6% vs. 6.9%) [19].

Modality-specific complications and contraindications further shape patient selection. For PVTT-directed RT, key constraints include radiation-associated liver injury, interval hepatic deterioration, and viral hepatitis reactivation (particularly HBV), which necessitate proactive assessment and management; practical contraindications include decompensated cirrhosis, uncontrolled hepatitis activity, or inadequate non-irradiated liver volume [32,33]. HAIC introduces risks of hepatic decompensation in marginal reserve, cytopenia and infection risk, and catheter/arterial access-associated complications; it is practically contraindicated in poor reserve (high bilirubin, Child–Pugh B/C, inadequate FLR or poor ICG retention where used), uncontrolled infection, or technical inability to deliver infusion safely [34,35]. TACE-containing combinations (including triple therapy) carry a risk of post-embolisation hepatic insufficiency when portal flow is compromised, alongside TKI-related hypertension/proteinuria and immune-related adverse events; major portal obstruction without robust collateralisation, severe portal hypertension/recent variceal bleeding, or decompensation commonly limit suitability [36,37]. For systemic conversion strategies (TKI + PD-1 ± anti-VEGF), key issues include bleeding risk in portal hypertension (particularly with anti-VEGF), immune-related toxicities, and perioperative wound/bleeding concerns requiring appropriate washout; uncontrolled varices/high bleeding risk, significant autoimmune disease or uncontrolled infection, and poor performance status are typical barriers [38].

Finally, effectiveness is judged by OS and DFS/RFS, while acknowledging that stage mix strongly confounds cross-study comparison. RT has the highest comparative certainty: in Wei et al., 24-month OS was 27.4% vs. 9.4% and 24-month DFS was 13.3% vs. 3.3% for RT + surgery versus surgery alone, with recurrence events common but fewer with RT (66/82 vs. 75/82) [26]. In Li et al. (Cheng III), early recurrence was reduced with RT (6 months, 49.0% vs. 88.7%; 12 months, 77.0% vs. 97.7%) with improved short-term OS (1 year, 69.0% vs. 35.6%; 2 years, 20.4% vs. 0%) [23]. HAIC shows high ORR and strong matched long-term signals: in Hu et al. (PSM), 1/3/5-year OS was 97.5/81.9/69.3% vs. 67.3/33.9/28.2%, and matched RFS was 62.1/45.7/45.7% vs. 12.2/9.2/9.2%, with benefit most prominent in Vp3/4 and not statistically significant in Vp1/2, supporting stage-adapted selection and early response monitoring [21]. In branch PVTT, triple therapy achieved deep responses and substantially lower recurrence in retrospective comparison: Hou et al. reported recurrence of 11/33 (10.5%) in NASR versus 80/105 (76.2%) in the surgery resection cohort, alongside CR/PR, each 39.4%, and pCR, 27.2% [19]. In major PVTT conversion/salvage settings, resection is achievable in a subset and survival improves in converted patients, but recurrence remains common in non-pCR patients; in Jiao et al., median RFS after salvage surgery was ~11 months with 1- and 3-year RFS of 72.5% and 30.7%, while Hamaoka et al. similarly demonstrated a strong survival advantage among patients who reached hepatectomy [20,22].

In summary, modality choice aligns strongly with PVTT extent and deliverability. Branch PVTT (Cheng I–II) is best positioned for embolisation-based systemic combinations capable of deep response (including pCR), whereas major PVTT (Cheng II–III and beyond; Vp3/Vp4) often prioritises PVTT-directed RT, HAIC, and/or systemic conversion with early response reassessment. A clinically useful synthesis is therefore to treat neoadjuvant therapy as a staged pathway: match modality to PVTT anatomy and hepatic reserve, monitor both tumour and thrombus response early (with explicit non-response denominators and attrition to surgery), and transition promptly to resection when technically feasible while avoiding prolonged ineffective treatment that risks losing the curative window. Where multimodality therapy is planned, sequencing may be guided by the dominant anatomy-driven constraint. In proximal PVTT (Vp3/4; Cheng III/IV), initial treatment could prioritise thrombus control and preservation of portal flow (e.g., PVTT-directed radiotherapy and/or HAIC), whereas in distal/branch PVTT, first-line neoadjuvant therapy may reasonably focus on reducing intrahepatic tumour burden using embolisation-based and systemic regimens. Subsequent components can then be introduced in a response-adapted manner once liver function and resectability have been maintained or restored.

The principal limitation of this review is the underlying evidence base. Only one included study was a randomised controlled trial, whereas the remainder comprised retrospective cohort studies and a non-randomised comparative study. Consequently, confounding by indication, selection bias, and incomplete adjustment for baseline imbalance are likely, consistent with ROBINS-I assessments, which found several retrospective studies to be at serious or critical risk of bias. Second, postoperative management was not uniformly reported. Adjuvant or maintenance therapies were inconsistently described across studies, and, where protocolised postoperative treatment differed between groups, the observed recurrence and survival differences cannot be attributed to the neoadjuvant component alone with confidence. Third, clinical heterogeneity limited direct cross-modality comparison. Neoadjuvant regimens varied substantially, were applied across different PVTT extents and staging systems, and were often assessed using different response and thrombus endpoints, precluding definitive conclusions on comparative effectiveness. Finally, external validity is limited. All included studies were conducted in Eastern Asian populations in which HBV-related HCC is common. This case mix differs from many Western cohorts with higher proportions of HCV, alcohol-related liver disease, and NAFLD/NASH.

Looking ahead, three priorities for future research emerge. First, the next generation of studies should move beyond “combination versus single modality” comparisons to include head-to-head evaluations of combination regimens (combination vs. combination), using harmonised PVTT classification and intention-to-treat reporting (including attrition to resection) to clarify comparative effectiveness and safety. Second, because multimodality pathways are inherently sequential, prospective studies should explicitly test treatment sequencing (e.g., PVTT-directed local control first versus systemic/arterial therapy first), ideally with response-adapted designs stratified by PVTT extent and hepatic reserve to minimise loss of the surgical window. Third, standardised PVTT response assessment is needed that links radiological metrics (extent, portal flow, and viable thrombus) to pathological findings at resection; quantifying radiology–pathology discordance (as suggested by salvage cohorts) may refine endpoints that best predict recurrence. Across all three priorities, multicentre recruitment that includes Western, aetiologically diverse populations will be important to strengthen external validity.

## 5. Conclusions

HCC with PVTT is a high-risk phenotype in which neoadjuvant therapy should be considered with radiological response, deliverability, and preservation of a curative surgical window. Across studies, outcomes and treatment aims were closely linked to the extent of portal vein involvement, enabling conversion to resection in selected patients. Primary tumour and PVTT responses did not consistently align, and radiology may not fully capture the thrombus treatment effect, supporting a dual-compartment assessment strategy. Overall, these data support a stage- and response-adapted approach—matching the initial modality to the dominant anatomical constraint, monitoring early for tumour and PVTT control, and proceeding to resection when feasible. Interpretation remains limited by predominantly non-randomised evidence, heterogeneous endpoints, and variable postoperative therapy reporting, with restricted generalisability from HBV-endemic Eastern Asian cohorts. Future prospective work could therefore prioritise head-to-head combination vs. combination evaluations, sequencing studies, and standardised radiological–pathological PVTT endpoints in multicentre, aetiologically diverse populations.

## Figures and Tables

**Table 1:** Risk of bias assessments based on the ROBINS-1 assessment tool for retrospective cohort studies.

Author	Domain 1 Score	Domain 2 Score	Domain 3 Score	Domain 4 Score	Domain 5 Score	Domain 6 Score	Domain 7 Score	Overall SCORE
Hou [19]	Low	Moderate	Low	Low	Low	Low	Low	Moderate
Jiao [20]	Critical	Serious	Low	Low	Low	Low	Low	Critical
Hu [21]	Low	Serious	Moderate	Low	Low	Low	Low	Serious
Hamaoka [22]	Low	Low	Moderate	Serious	Low	Moderate	Low	Serious
Li [23]	Low	Low	Low	Low	Low	Low	Low	Moderate
Yamamoto [24]	Critical	Low	Critical	Low	Low	Low	Moderate	Critical

**Table 2:** Risk of bias assessment based on the ROB-2 assessment tool for randomised controlled trials.

Author	Domain 1 Score	Domain 2 Score	Domain 3 Score	Domain 4 Score	Domain 5 Score	Overall Score
Wei [26]	Low	Low	Low	Low	Low	Low

**Table 3:** Main characteristics of included studies (TKIs, Tyrosine Kinase Inhibitors; HAIC, Hepatic Artery Infusion of Chemotherapy; 3D-CRT, 3D-conformal Radiotherapy; TACE, Trans-Arterial Chemo-Embolisation.

Study	Year	Study Type	Intervention	Total Population	Outcome of Interest
Hou [19]	2024	Retrospective Cohort	TKIs/PD-1 Antibodies/TACE	138	OS, RFS
Jiao [20]	2023	Retrospective Cohort	TKIs/PD-1 Antibodies	40	OS
Hu [21]	2023	Retrospective Cohort	HAIC	120	OS, RFS
Wei [26]	2019	Randomised Controlled Trial	3D-CRT	164	OS, RFS
Hamaoka [22]	2017	Retrospective Cohort	HAIC + 3D-CRT	50	OS
Li [23]	2016	Non-Randomised Comparative Study	3D-CRT	89	OS, RFS
Yamamoto [24]	2015	Retrospective Cohort	TACE	372	OS

## Data Availability

The data that support the findings of this study are available from the corresponding author, [PR], upon reasonable request.
